# Factors affecting outcome of longer regimen multidrug-resistant tuberculosis treatment in West Java Indonesia: A retrospective cohort study

**DOI:** 10.1371/journal.pone.0246284

**Published:** 2021-02-08

**Authors:** Arto Yuwono Soeroto, Chica Pratiwi, Prayudi Santoso, Bony Wiem Lestari

**Affiliations:** 1 Division of Respirology and Critical Care Medicine, Department of Internal Medicine, Faculty of Medicine Universitas Padjadjaran, Dr. Hasan Sadikin General Hospital, Bandung, West Java, Indonesia; 2 Department of Internal Medicine, Faculty of Medicine Universitas Padjadjaran, Dr. Hasan Sadikin General Hospital, Bandung, West Java, Indonesia; 3 Department of Public Health, Faculty of Medicine Universitas Padjadjaran, Bandung, West Java, Indonesia; 4 TB-HIV Research Center, Faculty of Medicine Universitas Padjadjaran, Bandung, West Java, Indonesia; 1. IRCCS Neuromed 2. Doctors with Africa CUAMM, ITALY

## Abstract

**Background:**

Multidrug-resistant tuberculosis had high treatment failure and mortality. Success rate of treatment currently 56% at global level, 48% in Indonesia and 36% in West Java province, the most populated province and surround Jakarta, the capitol of Indonesia.

**Objective:**

This study aimed to evaluate factors affecting success of multidrug-resistant tuberculosis treatment in patients using longer treatment regimen in West Java Indonesia.

**Methods:**

This was a retrospective cohort study of multidrug-resistant tuberculosis patients treated with longer regimen at Hasan Sadikin General Hospital from January 2015 to December 2017. Potential risk factors associated with the treatment outcome were analyzed using multiple logistic regression.

**Results:**

A total of 492 patients were enrolled during the study period. Fifty percents multidrug-resistant tuberculosis patients had successful treatment outcome. Age ≤45 years, male, normal body mass index, no previous tuberculosis treatment, culture conversion ≤2 months, acid fast bacilli sputum smear ≤+1 were independent factors associated with increased treatment success. Sputum culture conversion ≤2 months was the major factor affecting successful outcome (RR 2.79; 95% CI: 1.61–4.84; p-value<0.001). Human Immunodeficiency Virus infection, chronic kidney disease, and cavitary lesion were independent risk factors for unfavourable outcome.

**Conclusion:**

Age, gender, body mass index, tuberculosis treatment history, time of sputum conversion, acid fast bacilli sputum smear, HIV infection, chronic kidney disease, and cavitary lesion can be used as predictors for longer multidrug-resistant tuberculosis treatment regimen outcome.

## Introduction

Multidrug-resistant tuberculosis (MDR-TB) is a condition in which *Mycobacterium tuberculosis* is resistant to at least isoniazid and rifampicin. MDR-TB cases is increasing every year globally. There were 160,684 new cases of MDR-TB in 2017 and 186,772 new cases in 2018. MDR-TB was detected in 3.4% of new TB cases and 18% of previously treated cases [1]. The burden of MDR-TB varies among countries where India (27%), China (14%), and The Russian Federation (9%) were the top three countries contributed to the largest share of the global burden [1]. MDR-TB was also a growing problem in low incidence countries. TB surveillance in the European Union or European Economic Area (EU/EEA) countries in 2014 showed that 4.0% of TB cases were MDR-TB (2.2% of all notified TB cases) as identified by the drug susceptibility testing (DST) [2]. While the prevalence of MDR-TB from TB cases undergoing DST was 1.2% in the US, 1.3% in Canada, and 2% in Australia [2].

Indonesia is one of 20 main countries with the highest burden of MDR-TB. WHO (World Health Organization) estimates in 2018, cases of MDR-TB in Indonesia were about 24,000 cases per year including 2.4% of new TB (Tuberculosis) cases and 13% of re-treatment TB cases with treatment success rate of 48% [1]. West Java is one of provinces in Indonesia with most of MDR-TB cases. Based on Ministry of Health data, there were 1.566 new cases in 2018 and 2.073 cases in 2019. There only 45–50% MDR-TB cases that got treated, with 36% success rate in West Java [3]. A study in West Java showed that living in rural area was associated with delay to MDR-TB diagnosis and treatment initiation, with median time to diagnosis was 14 days and 25 days for treatment [4]. However, no study has been performed in Indonesia to evaluate risk factors associated with MDR-TB treatment outcome, especially in West Java.

MDR-TB has a high treatment failure rate and death. In 2018, WHO data showed that of those with MDR-TB in the world, only 32% of cases underwent therapy, with a success rate of 56%. Researches conducted in countries with a high burden of MDR-TB found different factors that affect MDR-TB treatment outcomes. In China, MDR-TB patients who smoke, drink, have ofloxacin resistance, or a high smear grade are less likely to respond to treatment. Predictors of unsuccessful treatment in Egypt were delayed culture conversion, moderate or extensive lung affection, and diabetes mellitus. India found different factors affected poor treatment outcome like baseline BMI <18, seven missed doses in intensive phase and continuation phase, cavitary disease, prior treatment episodes, longer duration and more episodes of treatment, any weight loss during treatment, males and additional resistance to first line drugs (Ethambutol, Streptomycin) [5–7]. Some socioeconomic determinants had also been associated with failure of MDR-TB treatment such as low education, low income, alcohol abuse, unemployment, and lack of health insurance [8, 9].

MDR-TB treatment, especially the longer regimen is often associated with poor treatment outcome [10]. However, longer regimens are still the main regimen used in MDR-TB endemic countries in Asia and Africa including Indonesia, while shorter regimens at present are only applied in 20 countries, and only given to patients who do not have contraindications [11].

As studies that evaluate the combination of longer regimens have not been evenly conducted in countries with a high burden of TB and there have not been many studies on the factors that influence the success of MDR-TB using longer treatment regimen [1]. Therefore, in this study, we aimed to identify factors that associated with successful treatment outcome in a high burden MDR-TB setting in Indonesia.

## Materials and methods

### Design and setting

This was a retrospective cohort study conducted in the Hasan Sadikin General Hospital in Bandung. This hospital is a tertiary referral hospital that provides treatment for drug-resistant TB patients. Data was collected at PMDT (Programmatic Management of Drug-resistant TB) clinic from December 2019 to January 2020. Patients’ information were inputted into e-TB Manager database, which is a system for recording and reporting MDR-TB cases. This study had received approval from the Health Research Ethics Committee of Hasan Sadikin General Hospital number LB.02.01/X.6.5/356/2019. Patient’s data from medical records were de-identified and analyzed anonymously.

### Study population

The study population was all MDR-TB patients aged 18 years old or more, who received longer treatment regimens from January 2015 to December 2017. Longer regimen used in our hospital were combination of fluoroquinolone (levofloxacin or moxifloxacin), aminoglycosides injection (kanamycin or capreomycin), ethionamide, cycloserine, and pyrazinamide. Patients were followed up until treatment complete, with minimum duration was 18 months.

### Variables

This study analyzed 13 independent variables that might affect outcome of MDR-TB therapy. Age, sex, smoking status, BMI (Body Mass Index), HIV (Human Immunodeficiency Virus), DM (diabetes mellitus), CKD (Chronic Kidney Disease), anemia, history of TB medication, time of sputum conversion, quantity result of Xpert MTB/RIF, AFB (Acid Fast Bacilli) smear, and cavity in baseline thorax x-ray were categorized and presented in proportions and percentages.

The outcome of study was success and unfavorable outcomes. A successful treatment was defined as patient who fulfill recovery criteria or completed treatment. Unfavorable outcomes were defined as a combination of dropout rates, failure of therapy, and death. Success criteria and unfavorable outcomes used in this research were in accordance with WHO criteria [6].

### Statistical analysis

Univariate analysis was performed with Chi-square or Fisher’s exact test. Multivariate logistic regression was performed for variables with p<0.25 based on univariate analysis. Stata version 14.2 for Windows was adopted for statistical analysis. Two-tailed p≤0.05 was considered as statistically significant. Variables that had missing data were excluded from multivariate analysis. Patients who moved to another health facility and treated irregularly were excluded from the study.

## Results

**Fig 1:**
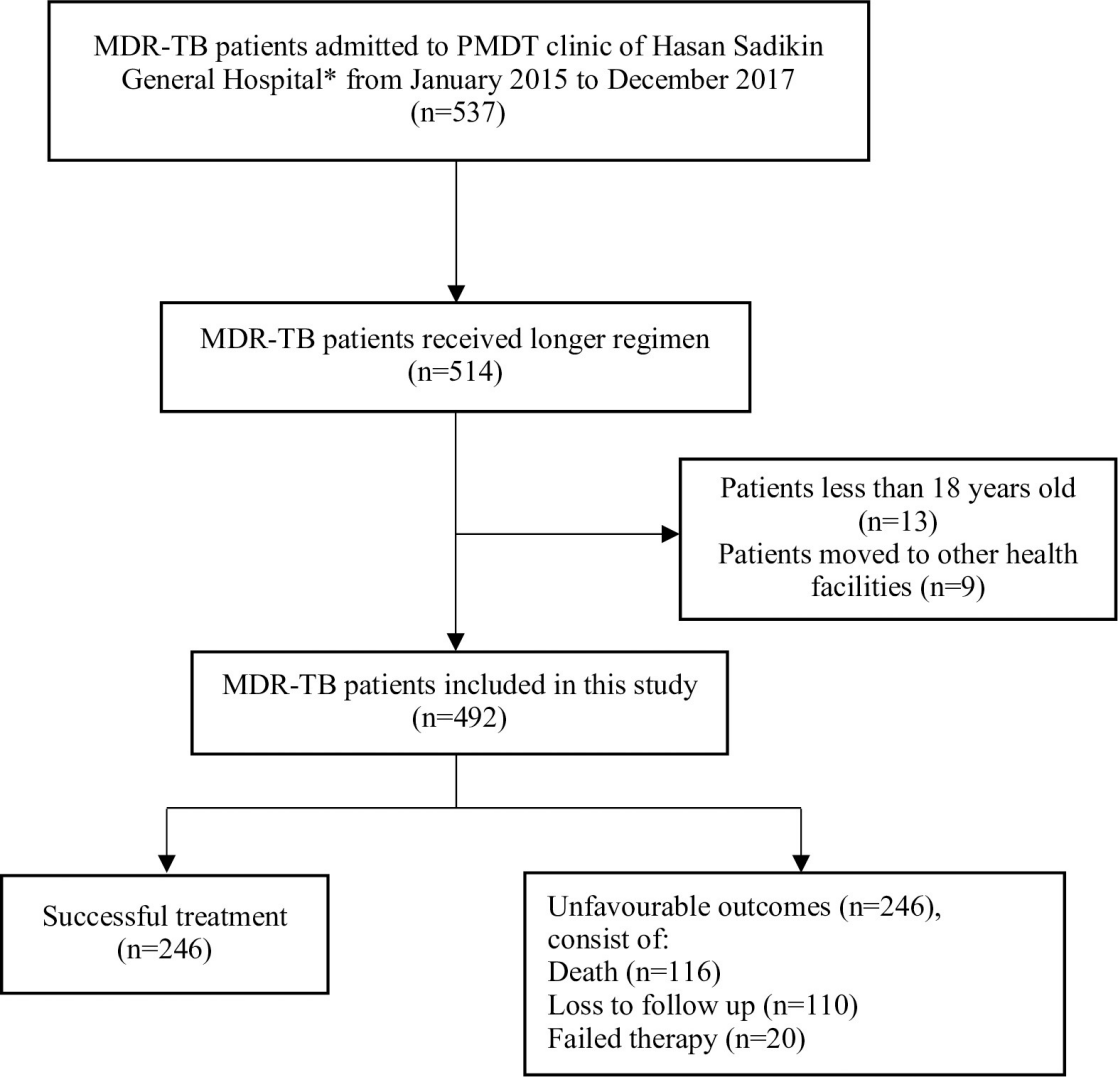
Flow chart of study participants. MDR-TB: Multidrug-resistant TB, PMDT: Programmatic Management of Drug-resistant TB. * Tertiary referral hospital that provides treatment for drug-resistant TB patients.

**Fig 2:**
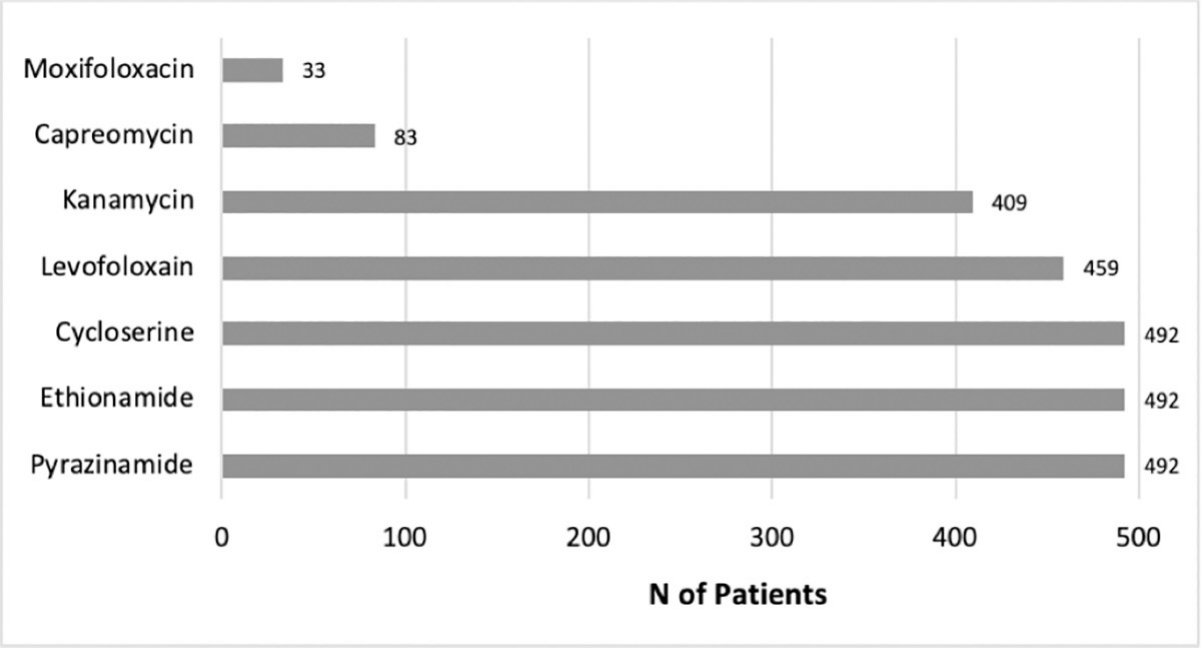
Longer regimen combination used for MDR-TB patients. MDR-TB: Multidrug-resistant TB, N: Number.

Fig 1 showed that there were 537 MDR-TB patients undergoing treatment from January 2015 to December 2017. Five hundred fourteen patients received longer regimens but only 492 patients were fulfilled eligibility criteria and included in this study. Kanamycin-levofloxacin based were the most frequent combination used for MDR-TB patients followed by capreomycin-levofloxacin, capreomycin-moxifloxacin, and kanamycin-moxifloxacin as described by Fig 2. Pyrazinamide, cycloserine, and ethionamide were also added to all combinations.

Overall, the successful treatment rate was 50%. The baseline characteristics were summarized in Table 1. Univariate analysis from Table 2 showed only smoking was not statistically significant between groups with p = 0.415, therefore smoking was not included in multiple logistic regression. Xpert MTB/RIF was not included in multivariate analysis regardless of p<0.05 because of incomplete data.

**Table 1:** Characteristics of MDR-TB patients (n = 492).

Characteristics	n (%)
Age (years)	
≤ 45	335 (68)
> 45	157 (32)
Sex	
Male	268 (54.5)
Female	224 (45.5)
Current Smoking Status	
Yes	271 (55)
No	221 (45)
BMI (kg/m^2^)	
Underweight	321 (65.3)
Normal	143 (29)
Overweight	16 (3.3)
Obese	12 (2.4)
Anemia	
Yes	252 (51.2)
No	240 (48.8)
HIV	
Yes	15 (3)
No	477 (97)
Diabetes mellitus	
Yes	76 (15.4)
No	416 (84.6)
CKD	
Yes	59 (12)
No	433 (88)
History of TB Medication	
No previous treatment	25 (5)
Relapse	167 (34)
Failed	216 (43.9)
Loss to follow up	84 (17.1)
Time of Sputum Conversion	
≤ 2 months	413 (84)
> 2 months	79 (16)
Xpert MTB/RIF	
High	69 (14)
Medium	102 (20.8)
Low	73 (14.8)
Very low	14 (2.9)
Missing data	234 (47.5)
AFB Smear	
≤ +1	180 (36.6)
> +1	312 (63.4)
Cavity	
Yes	214 (43.5)
No	278 (56.5)

BMI: Body Mass Index; HIV: Human Immunodeficiency Virus; CKD: Chronic Kidney Disease; TB: Tuberculosis; AFB: Acid-Fast Bacilli.

**Table 2:** Univariate analysis of factors affecting outcome of longer regimen MDR-TB treatment.

Variables	Success	Unfavorable outcomes	p-value*	RR (95% CI)
	n (%)	n (%)		
Age (years)				
≤ 45	178 (53.1)	157 (46.9)	0.042	1.23 (0.99–1.51)
> 45	68 (43.3)	89 (56.7)		
Sex				
Male	153 (57.1)	115 (42.9)	0.001	1.37 (1.14–1.66)
Female	93 (41.5)	131 (58.5)		
Current Smoking Status				
Yes	140 (51.7)	131 (48.3)	0.415	1.08 (0.90–1.29)
No	106 (48.0)	115 (52.0)		
BMI (kg/m^2^)				
Underweight	133 (41.4)	188 (58.6)		Reference
Normal	89 (62.2)	54 (37.8)	<0.001	1.50 (1.25–1.80)
Overweight	14 (87.5)	2 (12.5)	<0.001	2.11 (1.68–2.65)
Obese	10 (83.3)	2 (16.7)	<0.001	2.01 (1.51–2.67)
Anemia				
Yes	99 (39.3)	153 (60.7)	<0.001	0.64 (0.53–0.77)
No	147 (61.3)	93 (38.8)		
HIV				
Yes	1 (6.7)	14 (93.3)	<0.001	0.13 (0.02–0.86)
No	245 (51.4)	232 (48.6)		
Diabetes mellitus				
Yes	27 (35.5)	49 (64.5)	0.006	0.67 (0.49–0.93)
No	219 (52.6)	197 (47.4)		
CKD				
Yes	12 (20.3)	47 (79.7)	<0.001	0.38 (0.22–0.63)
No	234 (54.0)	199 (46.0)		
History of TB Medication				
No previous treatment	16 (64.0)	9 (36.0)	<0.001	3.36 (1.98–5.71)
Relapse	127 (76.0)	40 (24.0)	<0.001	3.99 (2.55–6.25)
Failed	87 (40.3)	129 (59.7)	0.002	2.11 (1.32–3.38)
Loss to follow up	16 (19.0)	68 (81.0)		Reference
Time of Sputum Conversion				
≤ 2 months	238 (57.6)	175 (42.4)	<0.001	5.69 (2.93–11.03)
> 2 months	8 (10.1)	71 (89.9)		
Xpert MTB/RIF				
High	9 (13.0)	60 (87.0)		Reference
Medium	44 (43.1)	58 (56.9)	<0.001	3.31 (1.73–6.33)
Low	54 (74.0)	19 (26.0)	<0.001	5.67 (3.04–10.59)
Very low	13 (92.9)	1 (7.1)	<0.001	7.12 (3.81–13.32)
AFB Smear				
≤ +1	161 (89.4)	19 (10.6)	<0.001	3.28 (2.72–3.96)
> +1	85 (27.5)	227 (72.8)		
Cavity				
Yes	48 (22.4)	166 (77.6)	<0.001	0.31 (0.24–0.41)
No	198 (71.2)	80 (28.8)		

*p<0.05 considered statistically significant. Univariate analysis was performed using Chi-Square, BMI: Body Mass Index; HIV: Human Immunodeficiency Virus; CKD: Chronic Kidney Disease; TB: Tuberculosis; AFB: Acid-Fast Bacilli; RR: Relative Risk; CI: Confidence Interval.

### Multiple logistic regression

Multivariate analysis with logistic regression from 11 factors showed that anemia and DM had no significant effect on the successful treatment (Table 3). Age ≤45 years, male, normal BMI, no previous tuberculosis treatment, culture conversion ≤2 months, AFB sputum smear ≤+1 were independent factors associated with increased treatment success. Time of sputum conversion in the first two months was the highest factor that increase the chances of success with RR = 2.79 and p<0.001. HIV, CKD, and cavitary lesion were independent risk factors for unfavourable outcome. HIV is a major factor that decreased success rate with 86.1% reduction (RR = 0.14 and p = 0.017).

**Table 3:** Multivariate analysis of factors affecting outcome of longer regimen MDR-TB treatment.

Variables	Initial Model		Final Model	
	Crude RR (95% CI)	p-value	Adjusted RR (95% CI)	p-value
Age (years)				
≤ 45	1.18 (1.01–1.38)	0.034	1.19 (1.02–1.38)	0.028
> 45	Reference		Reference	
Sex				
Male	1.23 (1.07–1.43)	0.006	1.24 (1.06–1.44)	0.005
Female	Reference		Reference	
BMI (kg/m^2^)				
Underweight	Reference		Reference	
Normal	1.18 (1.02–1.36)	0.026	1.21 (1.05–1.39)	0.007
Overweight	1.52 (1.13–2.05)	0.006	1.54 (1.15–2.07)	0.004
Obese	1.43 (1.01–2.02)	0.043	1.45 (1.02–2.05)	0.039
Anemia				
Yes	0.99 (0.77–1.03)	0.109		
No	Reference			
HIV				
Yes	0.15 (0.03–0.75)	0.021	0.14 (0.03–0.70)	0.017
No	Reference		Reference	
Diabetes mellitus				
Yes	0.96 (0.78–1.18)	0.695		
No	Reference			
CKD				
Yes	0.63 (0.41–0.96)	0.031	0.62 (0.41–0.94)	0.024
No	Reference		Reference	
History of TB Medication				
No previous treatment	2.20 (1.40–3.48)	0.001	2.20 (1.40–3.47)	0.001
Relapse	2.20 (1.50–3.23)	<0.001	2.20 (1.50–3.22)	<0.001
Failed	1.75 (1.19–2.56)	0.004	1.77 (1.21–2.58)	0.003
Loss to follow up	Reference		Reference	
Time of Sputum Conversion				
≤ 2 months	2.77 (1.60–4.81)	<0.001	2.79 (1.61–4.84)	<0.001
> 2 months	Reference		Reference	
AFB Smear				
≤ +1	1.96 (1.62–2.37)	<0.001	1.99 (1.64–2.40)	<0.001
> +1	Reference		Reference	
Cavity				
Yes	0.60 (0.48–0.76)	<0.001	0.59 (0.47–0.75)	<0.001
No	Reference		Reference	

Multivariate analysis using logistic regression. BMI: Body Mass Index; HIV: Human Immunodeficiency Virus; CKD: Chronic Kidney Disease; TB: Tuberculosis; AFB: Acid-Fast Bacilli; RR: Relative Risk; CI: Confidence Interval.

## Discussion

This was the first retrospective cohort study evaluating multiple factors that influence the success of MDR-TB treatments using longer regimens at the MDR-TB Clinic Hasan Sadikin General Hospital Bandung West Java Indonesia. This study population was 492 patients and analyzed the role of thirteen variables. The results showed that from thirteen variables studied, there were nine factors that influenced the successful treatment rate.

MDR-TB is more common in patients aged <45 years. This results are consistent with MDR-TB epidemiologic study in Indonesia, which showed that 61.6% of MDR-TB patients were in the productive age (18–50 years) [12]. However, patients aged <45 years had success rate 1.19 times higher than older age. Comorbid diseases and atypical symptoms increase the difficulty in diagnosis and management of patients in older age [13]. Physiological and biological changes in elderly also could impair microbial clearance mechanism and reduce cellular immune response [14]. MDR-TB patients over 45 years tend to have failed treatment two times higher and even three times higher in patients aged over 65 years [13]. Older age is associated with lower drug absorption due to reduced intestinal function and motility. Adverse effects and drug interactions are also higher due to reduced liver and kidney function for drug elimination [15]. Retrospective study in Italian referral hospital found that having risk factors of TB (DM, malnutrition, alcohol consumption, and immunosuppressive therapy) and cavities on chest x-ray were predictors of adverse events of TB in elderly [14].

A study conducted in China in 2018 found 73.65% of MDR-TB patients were male. In addition to the high risk of exposure due to social interactions in men, hormonal factors also play a role in the body’s defense mechanism against TB. Estradiol increases the response of T-helper 1 lymphocytes, interferon gamma (IFN-ỿ), and tumor necrosis factor-alpha (TNF-α) that play a role in controlling tuberculosis infection. Estradiol also plays a role in the activation of macrophages, while testosterone plays a role in the activation and motility of neutrophils. The role of neutrophils in the pathology of tuberculosis is still less investigated [7, 13, 16, 17]. Our study found that male were 1.24 times to have successful treatment compared to women with p = 0.005. Studies in Africa, Bangladesh, and Syria found that women were more difficult to reach health services because of cultural barriers and low levels of education [18]. However, this study did not evaluate further regarding education, culture, and socioeconomics role.

TB infection increases the anabolic process, accompanied by reduced appetite and increased nutrient malabsorption. YY peptide hormone secreted by the distal small intestine and large intestine will increase in the presence of TB infection. This hormone plays a role in regulating appetite and absorption of nutrients [19]. This study found that MDR-TB patients with normal BMI have a 1.21 times higher chance of successful treatment compared to underweight. Studies in India showed that BMI <18 kg/m^2^ increases the risk failed treatment by 1.64 times (95% CI 1.28–2.11; p<0.001) [7]. Malnutrition interferes with cell-mediated immunity and increases the risk of drug-induced liver injury (DILI). Malnutrition also interferes with the pharmacokinetics of drugs, including anti-TB drugs, thereby reducing drug levels in the blood and decreasing the therapeutic effect of the drug [20].

In this study, HIV is the main factor that reduces the chance of successful therapy (86.1%) with p = 0.017. Not only for MDR-TB patients, HIV positive is also associated with therapy failure among drug-sensitive TB [21]. Cell-mediated immunity, specifically CD4 T lymphocytes, plays an important role in controlling TB infection. HIV patients with low CD4 T lymphocyte levels will increase the risk of severe TB infection and treatment difficulties [22]. In a systematic literature review and meta-analysis study in Sub-Saharan Africa, it was found that the mortality rate of MDR-TB with HIV up to 18.1% [23]. MDR-TB patients with HIV have many drugs to take so that drug interactions are higher. This also causes decreasing medication adherence and greater side effects [24]. Microbiological confirmation is also difficult to obtain in HIV patients which causes delays in handling MDR-TB [22, 25].

CKD is other comorbid that can reduce successful treatment rate (RR 0.62; p = 0.024). Patients with CKD have a weak immune system due to chronic inflammatory conditions [26]. Studies conducted in Taiwan found that CKD can increase severe drug reactions and mortality MDR-TB patients by 3.65 times (95% CI = 1.71–7.76). MDR-TB patients with CKD experience side effects more frequent and more progressive decline in kidney function [26, 27].

Primary MDR-TB patients have a higher chance of successful treatment 2.20 times compared to loss to follow-up. A study conducted in Malaysia found that TB treatment history was the strongest risk factor for determining treatment outcomes (adjusted OR 4.87; 95% CI: 2.84–8.38; p = 0.001). A history of TB treatment will increase the risk for large amounts of drug resistance. Special attention must be given for factors that underlie the incidence of loss to follow-up, so it does not reoccur in subsequent treatments [28].

Time of sputum conversion ≤2 months is a major factor that increase the chances of success based on multivariate analysis (RR 2,79; 95% CI 1,61–4,84; p-value = <0.001). Sputum culture has an important role in evaluating the treatment response [5, 6]. A study conducted in China also showed that time of sputum conversion in the first two months increased the successful treatment rate by 2.88 times with 95% CI: 1.11–7.45 [29]. Longer sputum conversion describes more drugs to which *M*. *tuberculosis* resistant. The effectiveness of second-line anti-TB drugs in MDR-TB patients is best evaluated in the first eight weeks of administration [30].

AFB smear ≤+1 increased the chance of successful treatment by 1,99 times (95% CI: 1,64–2,40, p = 0.001). This is consistent with a study in China that found MDR-TB patients with AFB smear >+1 had a lower tendency for culture conversion (Hazard ratio = 0.61; 95% CI: 0.41–0.91; p = 0.001). This is due to the greater bacterial load at a higher degree of positivity [5]. Fredejas, et al. (2018) found that semi-quantitative Xpert MTB/RIF assay equivalent to AFB smear, though this assay remains underutilised in many countries, including Indonesia. The degree of smear positivity is operator dependent so it has varying sensitivity. Therefore, WHO recommends Xpert MTB/RIF as the first test for TB diagnosis but does not replace the role of AFB smear [31]. AFB smear proved to play an independent risk factor in determining the successful treatment of MDR-TB.

High bacterial load is also described by the presence of cavitary lesions based on chest radiograph. Cavitary lesions decrease the chance of successful therapy by 40.5% (RR: 0.59; 95% CI: 0.47–0.75; p-value = <0.001). A study in China found that cavity increased the risk of poor treatment outcomes by 1.42 times (95% CI: 1.18–1.73; p-value = <0.001) [13]. Cavitary lesions are heterogeneous with a very high number of TB bacteria. Cavity, especially bilateral cavity, increases the relapse rate, resistance, and failure of therapy for MDR-TB patients due to the difficulty of drug penetration into cavity lesions [32].

The TB and DM interactions potentially cause adverse impact by increasing each other’s complications, making diagnosis and treatment more difficult, worsening disease course and outcome [33]. In our study, we found 15% of MDR-TB patients has DM at baseline before treatment initiation. The DM prevalence among MDR-TB patients is much higher compared to those with drug-sensitive TB [21, 34]. DM disrupts cellular immunity, chemotactic effect and phagocytosis of alveolar macrophage, interferon gamma, and also causes pulmonary microangiopathy and micronutrient deficiency. DM patients also have a high risk for impaired renal function and DILI [35]. Therefore, active screening of DM in patients with TB is highly recommended [33].

A study conducted in Korea found that successful treatment were lower in DM type 2 compared to without DM (36 vs. 47.2%, p = 0.002) [36]. Interestingly, another study stated that there was no difference between MDR-TB patients with DM and without DM with p = 0.054 [37].

The interaction of anti-TB drugs and anti-diabetes drugs is one of the factors that influence the success of therapy. Glycemic control is an important factor that can improve the therapeutic response and severity of MDR-TB patients with comorbid DM, but this study did not investigate role glycemic control in patients.This study also did not distinguish between the length of diagnosis of DM, which proved to have a role in TB severity [36, 38, 39].

Anemia is also a proven as a factor influence success in bivariate analysis (RR: 0.64; 95% CI: 0.53–0.78; p-value = <0.001), but is not an independent risk factor. The mechanism underlying anemia causes poor MDR-TB treatment outcomes are not well known. Iron deficiency anemia causes interference with T-cell-mediated immunity [40]. Irbah, et al found that anemia affect the time of sputum conversion, but the type and severity of anemia did not affect the delay in conversion [41].

Smoking status is the only risk factor that has not been proven to influence the success of MDR-TB treatment. Cigarette smoke affects the activity of macrophages and lymphocytes in the lung [42]. De-Boer, et al. (2014) states that changes in smoker immunity are influenced by the amount and duration of smoking. Smoking more than 20 cigarettes a day affects the success of MDR-TB treatments [43]. Our study did not evaluate the number of cigarettes in a day, the duration of smoking, the type of cigarette, and the history of smoking cessation during treatment.

Some limitation should be noted. This study used data from medical record so it could not evaluate other socioeconomic demographic variables and did not specifically analyze onset and severity of comorbid disease. Anti-TB drugs adverse effect was also not recorded.

## Conclusions

Male, age ≤45 years, normal BMI, sputum culture conversion ≤2 months, AFB smear ≤+1 and no previous TB treatment were factors independently increase treatment succes, with the major factor was sputum culture conversion ≤ 2 months. On the other hand HIV, CKD, and presence of cavitary lesions were risk factors of unfavourable outcome. These factors should always be considered in managing TB-MDR/RR patients with longer regimen.

## Supporting information

S1 File Data MDR-TB outcome. (ODS)
